# Prevalence of diabetic peripheral neuropathy in Africa: a systematic review and meta-analysis

**DOI:** 10.1186/s12902-020-0534-5

**Published:** 2020-04-15

**Authors:** Wondimeneh Shibabaw Shiferaw, Tadesse Yirga Akalu, Yeshamble Work, Yared Asmare Aynalem

**Affiliations:** 1 Department of Nursing, Institute of Medicine and College of Health Science, Debre Berhan University, P.O. Box 445, Debre Berhan, Ethiopia; 2 Department of Nursing, College of Health Science, Debre Markos University, P.O. Box 269, Debre Markos, Ethiopia; 3 Ethiopian Public Health Institute, Addis Ababa, Ethiopia

**Keywords:** Diabetic peripheral neuropathy, Diabetes mellitus, Systematic review, Meta-analysis, Africa

## Abstract

**Background:**

Diabetes mellitus (DM) is a global health care problem that can impose a substantial economic burden. Diabetic peripheral neuropathy (DPN) is a common microvascular complication of DM that increases the potential for morbidity and disability due to ulceration and amputation. Though there is a significant amount of variation in the primary studies on DM regarding the prevalence of DPN in Africa. Hence, this study was aimed to estimate the overall prevalence of DPN in DM patients in Africa.

**Methods:**

PubMed, Scopus, Google Scholar, African Journals OnLine, WHO African Library, and the Cochrane Review were systematically searched online to retrieve related articles. The Preferred Reporting Items for Systematic Review and Meta-Analysis (PRISMA) guidelines was followed. Heterogeneity across the included studies was evaluated by the inconsistency index (I^2^). Publication bias was examined by funnel plot and Egger’s regression test. The random-effect model was fitted to estimate the pooled prevalence of diabetic peripheral neuropathy among patients in Africa. The meta-analysis was performed using the STATA™ Version 14 software.

**Results:**

Twenty-three studies which includes 269,691 participants were included in the meta-analysis. The overall pooled prevalence of diabetic peripheral neuropathy was 46% (95% CI:36.21–55.78%). Based on the subgroup analysis, the highest prevalence of diabetic peripheral neuropathy in DM patients was reported in West Africa at 49.4% (95% CI: 32.74, 66.06).

**Conclusion:**

This study revealed that the overall prevalence of diabetic peripheral neuropathy is relatively high in Africa. Hence, DPN needs situation-based interventions and preventive strategies, which are specific to the country. Further meta-analysis is needed to identify associated factors for the occurrence of diabetic peripheral neuropathy.

## Background

Diabetes melllitus (DM) is a significant health concern for many countries in the world. According to the International Diabetic Federation’s (IDF) latest estimated data, about 425 million adults in 2017 were living with diabetes globally; by 2045, this number is projected to rise to 629 million. In Africa, by 2017, 39 million people were living with diabetes and by 2045, this number is projected to rise to 82 million [1]. Diabetes is also a significant cause of death around the world, with estimates being that in 2015 diabetes directly caused 1.6 million deaths worldwide [2]. Additionally, over the past decade, the prevalence of diabetes has risen faster in low and middle-income countries than in high-income countries [3].

Morbidity and mortality in patients with DM is mainly attributed to microvascular and macrovascular complications [4]. Diabetic peripheral neuropathy (DPN) is a common microvascular complication of DM that increases the potential for morbidity and disability due to ulceration and amputation [5]. DPN is an asymmetrical, sensorimotor polyneuropathy that is caused by metabolic and microvascular changes that result from long-term hyperglycaemia and metabolic disorder [6]. Moreover, DPN in its earliest stages leads to segmental demyelination, which subsequently results in delayed nerve conduction velocity [7].

The prevalence of DPN varies widely in the literature. This is due to differences in the diagnostic criteria employed, types of diabetes, the different methods of patient selection, and the sample size [8, 9]. However, it has been estimated that the prevalence of DPN is 8.4% in China [10], 48.1% in Sri Lanka [11], 29.2% in India [12], 56.2% in Yemen [9], 39.5% in Jordan [13], 71.1% in Nigeria [14], 16.6% in Ghana [15], and 29.5% in Ethiopia [16].

Peripheral nerve damage in diabetic patients is mostly irreversible. This has led health care professionals to focus on prevention as well as the identification of modifiable risk factors [17]. Studies suggest that numerous risk factors are responsible for DPN in DM patients including age, gender, duration of diabetes, the presence of microvascular complications, hypertension, area of residence, body mass index, glycated haemoglobin (HbA1c) level, alcohol intake, hyperglycaemia, cigarette smoking, physical inactivity, and marital status [12, 18–25].

Patients with DPN often suffer from the loss or absence of a protective sensation in the lower extremities leading to balance problems [26], risk of foot ulcerations [22], pain and disrupted sleep patterns [27], cardiovascular morbidity and mortality [19], reduced quality of life [28], and increased cost of treatment [29]. Previous studies have indicated that for those with high-risk diabetic neuropathy, proper management and early screening can minimize the occurrence of ulcers by 60% and amputations by 85% [30]. Moreover, different primary studies in Africa show the magnitude of DPN as a health issue in the region. However, these studies have demonstrated substantial variation regarding its prevalence. Therefore, this study was aimed to estimate the pooled prevalence of DPN in patients with DM in Africa. Findings from the current study would serve as a benchmark for policymakers to implement appropriate preventative measures and to alleviate the pressing problem of DPN.

## Methods

### Search strategy and database

To extract all relevant literature, electronic databases such as PubMed, Google Scholar, African Journals of OnLine, Scopus, Web of Science, WHO African Library, and the Cochrane Review were searched. In addition, a hand search of grey literature and other related articles were conducted to retrieve additional relevant articles. All electronic sources of information were searched for the period of January 1st, 2000 to August 22nd, 2019. The search was deployed using the following MeSH and free-text terms: “peripheral neuropathy”, “diabetic neuropathy”, “diabetic polyneuropathy”, “diabetes mellitus”, and “Africa”. Boolean operators like “AND” and “OR” were used to combine search terms.

### Eligibility criteria

Studies were included if they met the following criteria: (1) studies reported their outcome variable as prevalence of DPN, (2) articles were published in peer-reviewed journals or grey literature, (3) articles were published in English between 2000 to 2019, and (4) studies were conducted using an African population. Studies were excluded on any one of the following conditions: (1) the article was not fully accessible (i.e., the full text was not available) at the time of our search, (2) it was a duplicate citation, (3) the article had a sub-standard quality score per stated criteria, (4) the study was not relevant to DPN, (5) the study involved peripheral neuropathy not related to DM, and (6) the patients in the study had comorbidities of human immunodeficiency virus (HIV), tuberculosis, and/or chemotherapy.

### Selection and quality assessment

Data were extracted using a pre-piloted data extraction format prepared in Microsoft™ Excel. The extracted information from the literature included author names, year of publication, study area, study design, sample size, data collection year, data collection method, reported prevalence, and its 95% confidence interval. The data were extracted by three independent authors. The methodological and overall quality of each article was assessed by both authors based on the modified version of the Newcastle-Ottawa Scale (NOS) for cross-sectional studies [31]. Studies which scored ≥5 out of 10 points in three domains of the modified NOS for a cross-sectional study were included in the analysis [32]. Any disagreements at the time of data abstraction were reconciled by discussion and consensus, (Supplementary file 1).

### Statistical analysis

To estimate the pooled prevalence of DPN, a meta-analysis using the random effect DerSimonian and Laird model was deployed. Cochran’s Q chi-square statistics and I^2^ statistical test were conducted to assess the random variations between primary studies [33]. To minimize the random variations between the point estimates of the primary study, meta-regression, subgroup analyses, and sensitivity analysis were performed to investigate the sources of heterogeneity. Publication bias was assessed by visual inspection of a funnel plot. In addition, an Egger test was conducted and a *p* ≤ 0.05 was considered statistically significant for the presence of publication bias [34, 35]. The meta-analysis was performed using the STATA**™** version 14 statistical software for Windows™.

### Data synthesis and reporting

To estimate the pooled prevalence of DPN in patients with DM, this systematic review and meta-analysis was carried out using the Preferred Reporting Items for Systematic Reviews and Meta-Analyses (PRISMA) guidelines [36], and PRISMA checklist has been used. The weighted prevalence of DPN in patients with DM was presented using a forest plot.

## Results

### Search results

**Fig. 1:**
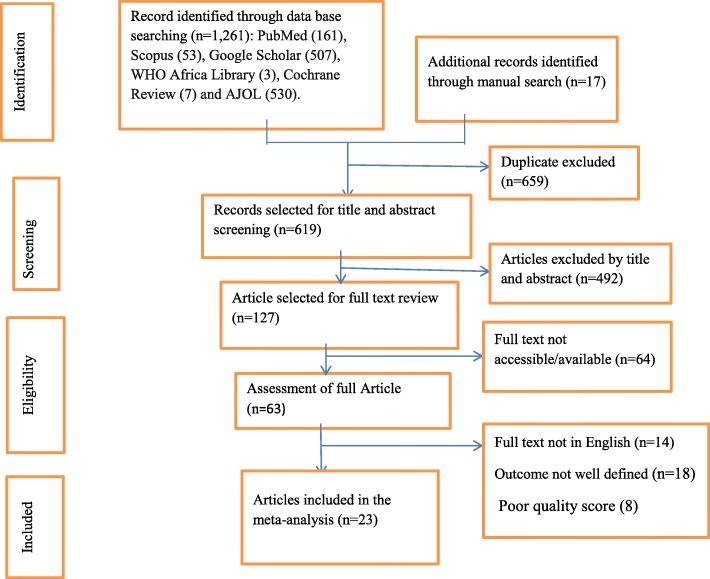
PRISMA flow chart for study selection

In total, 1278 studies were retrieved, of which, 1261 were found from six international databases and the remaining 17 were found through manual searches. The articles retrieved from the databases were as follows: PubMed (161), Scopus (53), Google Scholar (507), WHO African Library (3), Cochrane Reviews (7), and the African Journals OnLine (530). Of these papers, 659 duplicate records were identified and removed. From the remaining 619 articles, 492 articles were excluded after reading of titles and abstracts based on the pre-defined eligibility criteria. Finally, 127 full-text articles were read and assessed. After applying the pre-defined criteria and quality assessment, 23 articles were eligible for review and included in the final analysis (Fig. 1).

### Baseline characteristics of the included studies

A total of 23 studies with 269,691 study participants were included in this meta-analysis. Overall information regarding the prevalence of DPN in DM patients was obtained from various countries across Africa including 10 studies from Nigeria [14, 18–20, 22, 37–41], four from Ethiopia [16, 24, 42, 43], two from Cameroon [23, 44], two from Sudan [45, 46], two from Egypt [47, 48], and one each from Ghana [15], Uganda [49], and Tanzania [50]. The highest and lowest prevalence of DPN was 83.4% [22] and 7.5% [41] respectively, which were reported from Nigeria. Study sample sizes ranged from 50 to 524 participants. Moreover, based on the modified Newcastle-Ottawa quality score assessment, all 23 articles fulfilled the required quality score (Table 1).

**Table 1:** Characteristics of the studies included in the meta-analysis of diabetic peripheral neuropathy in patients with diabetes from Africa

Author	Publication year	Study Country, Continent	Study Design	Sample Size	Prevalence (95%(CI))	Data Collection year	Data Collection Method	Quality score
Adeniy et al. [38]	2015	Nigeria, West Africa	Cross-sectional	264	26.1 (20.8,31.4)	NA	Interview and physical examination	8
Amour et al. [50]	2019	Tanzania, East Africa	Cross-sectional	338	72.2 (67.3,77)	October 2017 to March 2018	Interview and physical examination	7
Awadalla et al. [45]	2017	Sudan, North Africa	Cross-sectional	424	68.2 (63.8,72.6)	NA	Interview, physical examination, and biochemical test	6
Bello et al. [19]	2019	Nigeria, West Africa	Cross-sectional	175	41.7 (34.4,49)	March 2014 to March 2015	Interview and physical examination	7
Ede et al. [22]	2018	Nigeria, West Africa	Cross-sectional	90	83.4 (75.7,91.1)	June 2016 to July 2017	Interview and physical examination	7
Gill et al. [42]	2008	Ethiopia, East Africa	Cohort study	105	41 (31.6,50.4)	NA	Interview and physical examination	8
Jarso et al. [43]	2011	Ethiopia, East Africa	Cross-sectional	384	48.2 (43.2,53.2)	NA	Interview and physical examination	8
Jember et al. [24]	2017	Ethiopia, East Africa	Cross-sectional	408	52.2 (47.1,57.3)	February 2016 to June 30, 2016	Record, interview, and physical examination	8
Khalil et al. [47]	2019	Egypt, North Africa	Cross-sectional	506	20 (16.5,23.5)	NA	Interview and physical examination	7
Kisozi et al. [49]	2017	Uganda, East Africa	Cross-sectional	288	29.4 (23.7,35)	December 1st, 2014 to March 31st, 2015	Interview and physical examination	7
Kuate-Tegueu et al. [44]	2015	Cameroon, Central Africa	Cross-sectional	306	33.3 (28,38.6)	February to June 2013	Interview and physical examination	7
Mba et al. [41]	2001	Nigeria,West Africa	Cross-sectional	286	7.5 (4.4,10.6)	NA	Interview and physical examination	8
Mohamed et al. [48]	2019	Egypt, North Africa	Cross-sectional	50	12 (2.99,21.01)	NA	Record and physical examination	7
Mohmad et al. [46]	2011	Sudan, North Africa	Cross-sectional	71	69 (58.2,79.7)	December 2006 to September 2008	Interview and physical examination	7
Ogbera et al. [40]	2015	Nigeria,West Africa	Cross-sectional	225	37 (30.7,43.3)	NA	Interview and physical examination	8
Oguejiofor et al. [51]	2019	Nigeria,West Africa	Cross-sectional	524	57.4 (53.2,61.6)	NA	Interview and physical examination	7
Ojieabu et al. [39]	2016	Nigeria,West Africa	Cross-sectional	167	59.2 (51.7,66.6)	2011–2012	Record review	8
Olamoyegun et al. [18]	2015	Nigeria,West Africa	Cross-sectional	92	69.6 (60.2,79)	January to May, 2013	Interview, physical examination, and biochemical analysis	6
Owolabi et al. [14]	2012	Nigeria, West Africa	Cross-sectional	277	71.1 (65.7,76.4)	February 2008 to March 2009	Record, interview, and physical examination	7
Tamba et al. [23]	2013	Cameroon, Central Africa	Cross-sectional	140	40 (31.9,48.1)	2000 to 2009	Record review	6
Ugoya et al. [37]	2006	Nigeria, West Africa	Cross-sectional	180	75 (68.7,81.3)	NA	Interview, physical examination, and biochemical analysis	7
Worku et al. [16]	2010	Ethiopia, East Africa	Cross-sectional	305	29.5 (24.4,34.6)	October 2008	Record review	7
Yeboah et al. [15]	2018	Ghana, West Africa	Case control	350	16.6 (12.7,20.5)	December 2012 to June 2013	Interview and physical examination	6

*NA* Not applicable

### Prevalence of DPN

**Fig. 2:**
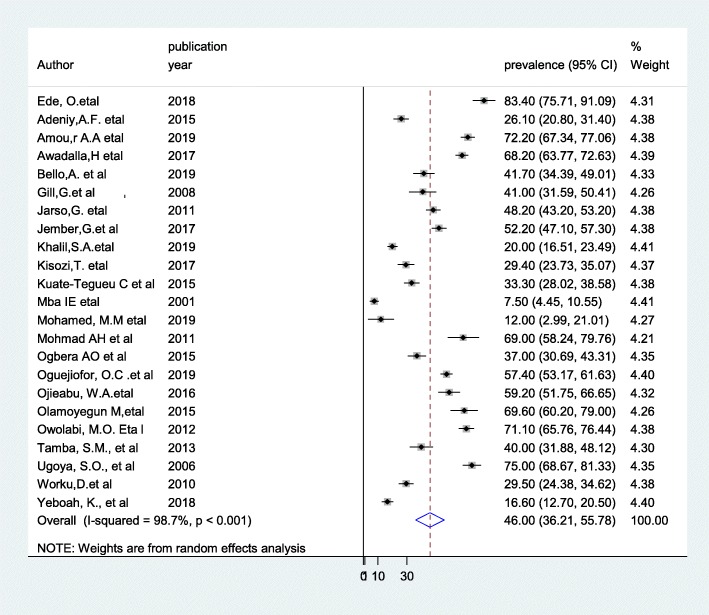
Forest plot showing the pooled prevalence of diabetic peripheral neuropathy

The result of this meta-analysis using a random effects model showed that the pooled prevalence of DPN was 46% (95% CI: 36.21–55.78) (Fig. 2) with significant heterogeneity being observed (*I*^2^ = 98.7%; *p* ≤ 0.001).

### Subgroup analysis

**Fig. 3:**
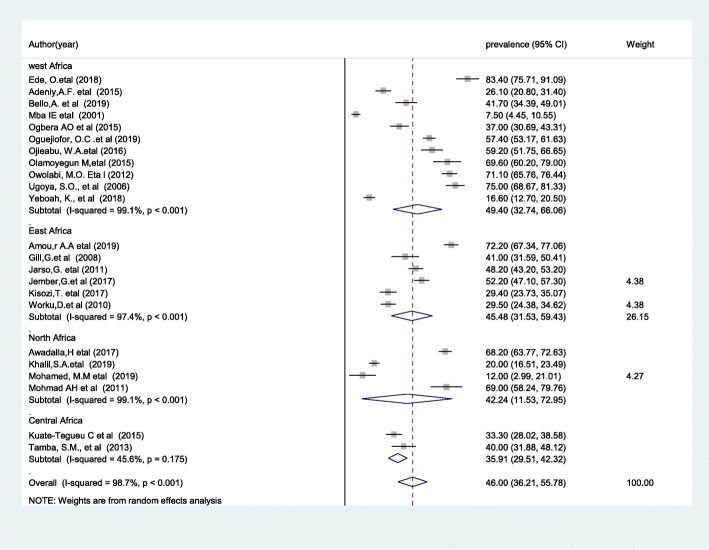
Forest plot of the subgroup analysis based on the country where the studies were conducted

The presence of significant heterogeneity among the primary studies required that we conduct subgroup analysis. As a result, in order to ascertain the sources of heterogeneity we deployed subgroup analysis using study area (geographic indicator) as the variable of interest. From this we found that the highest prevalence of DPN was observed in a study conducted in West Africa 49.4% (95% CI: 32.74, 66.06) (Fig. 3).

### Meta-regression analysis

To investigate the possible source(s) of variation across the included studies, we performed meta-regression analysis using publication year and sample size as covariates of interest. However, the result of this meta-regression analysis showed that both covariates were not significantly associated with the presence of heterogeneity, (Table 2).

**Table 2:** Meta-regression analysis for the included studies to identify the source(s) of heterogeneity

Covariate (source)	Coefficient	Standard error	*t*-value	*p*-value	95% CI
Publication year	0.014	0.295	0.05	0.962	− 0.607, 0.635
Sample size	− 0.001	0.010	− 0.10	0.924	−0.022, 0.020

### Publication bias

**Fig. 4:**
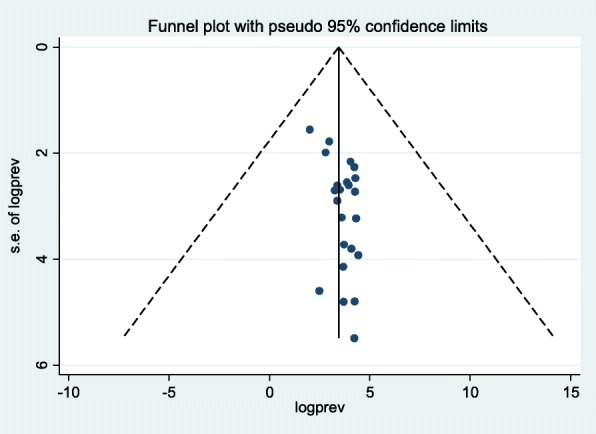
Funnel plot to determine the presence of publication bias among the 23 included studies

To identify the presence of publication bias, both a funnel plot and Egger’s test were performed. Visual inspection of the funnel plot showed an asymmetrical distribution, which indicated the presence of publication bias (Fig. 4). The finding of publication bias was affirmed following the Egger’s test (*p* = 0.024).

### Sensitivity analysis

**Fig. 5:**
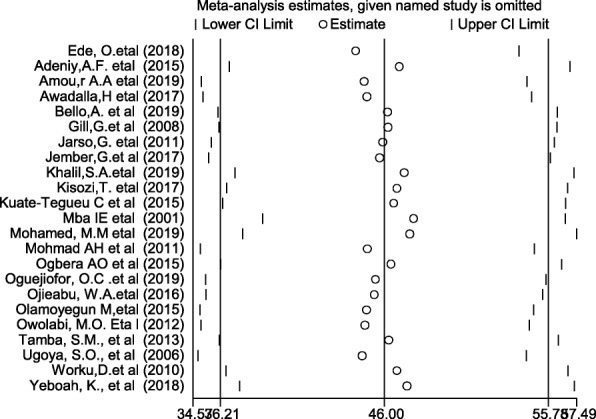
Sensitivity analysis of the 23 included studies

We conducted a sensitivity analysis to assess the effect of any individual study on the pooled effect size. Our analyses using a random effects model revealed that no single study affected the overall prevalence of DPN (Fig. 5).

## Discussion

In this systematic review and meta-analysis, the overall prevalence of DPN was 46% (95%CI: 36.21, 55.78). This finding was in line with a systematic review and meta-analysis conducted in Iran which yielded a prevalence of 53% [52]. In contrast, the prevalence found in our study was higher than a systematic review and meta-analysis conducted in developed countries, which reported a prevalence of 35.78% [53]. This variation could be the result of different diagnostic criteria for diabetic neuropathy, and early diagnosis and treatment in developed countries.

DPN prevalence varied greatly in the included studies, ranging from 7.5% [41] to 83.4% [22]. However, our subgroup analysis based on study area showed that the highest pooled prevalence of DPN was observed from studies done in West Africa (49.4%; 95% CI: 32.74, 66.06) and the lowest was observed in Central Africa (35.9%; 95% CI: 29.51, 42.32). This discrepancy could be explained by studies using different diagnostic criteria for diabetic neuropathy, the quality of the health care service, and the duration and severity of diabetes. The findings of this meta-analysis have implications for clinical practice. Specifically, estimating the pooled prevalence of DPN will indicate where preventative strategies are needed most and may reflect the quality of healthcare given to patients in a particular area.

This systematic review and meta-analysis was conducted based on PRISMA guidelines for literature reviews. In addition, publication bias was quantified using Egger’s regression statistical test, and NOS was used to assess the quality of the included studies. To the best of our knowledge this is the first study on the prevalence of DPN in DM patients from Africa which may be helpful for future researchers, public health practitioners, and health care policymakers.

This study was conducted with the use of a comprehensive search strategy to incorporate the studies involving African patients. All of the included studies were observational studies with high methodological quality based on NOS assessment. In addition, the inclusion of previously published studies that met our inclusion criteria further strengthened our meta-analysis. There are several limitations to this review which must be acknowledged and may inform future research. First, we only used English language articles although our target was the African content which could be in several other languages such as Spanish, French, or Portuguese. Also, our study was exclusively driven by hospital-based data, which reduced the community-based capture on this topic. Finally, we did not explore the predictors of DPN in DM patients.

## Conclusion

This study revealed that the overall prevalence of DPN was relatively high in Africa. Hence, African nations need to implement situation-based interventions and preventive strategies in order to try to curb this debilitating disease. In addition, policymakers and other concerned bodies need to give special attention to improve healthcare delivery for patients with DM to reduce the risk of DPN. Furthermore, further research is needed to identify associated factors for the development of DPN in patients with DM.

## Supplementary information

**Additional file 1: Supplementary file 1.** Methodological quality assessment of cross-sectional studies using the modified Newcastle-Ottawa Scale (NOS).

## Data Availability

The data analyzed during the current meta-analysis is available from the corresponding author on reasonable request.

## Abbreviations

CI: Confidence interval
DM: DIABETES mellitus
DPN: Diabetic peripheral neuropathy
NOS: Newcastle-Ottawa Scale
PRISMA: Preferred Reporting Items for Systematic Reviews and Meta-Analyses
