# Supplementary material for: Prevalence of diabetic peripheral neuropathy in Africa: a systematic review and meta-analysis
Source: BMC Endocr Disord. 2020 Apr 15;20:49. doi: 10.1186/s12902-020-0534-5 (PMC7158034; doi:10.1186/s12902-020-0534-5)
Supplement: Supplementary file 1 — Additional file 1: Supplementary file 1. Methodological quality assessment of cross-sectional studies using the modified Newcastle-Ottawa Scale (NOS). [file 12902_2020_534_MOESM1_ESM.docx]

**Methodological quality assessment**

Supplementary file 1: Methodological quality assessment of cross-sectional studies using modified Newcastle - Ottawa Scale (NOS)

| **First author, publication year** | Criteria | | | | | | | |  |
| --- | --- | --- | --- | --- | --- | --- | --- | --- | --- |
|  | **Selection** | | | | **Comparability** | | **Outcome** | |  |
|  | **Representativeness of the sample** | **Sample size** | **Non –responders** | **Ascertainment of exposure/risk factor** | **The study controls for the most important factor** | **The study control for any additional factor** | **Assessment of the outcome** | **Statistical test** | **Total score**  **(10)** |
| Adeniy,A.F. etal,2015 | **A*** | **A*** | **A*** | **A*** | **A*** | **B*** | A* | **A*** | **8** |
| Amou,r A.A etal 2019 | A* | **B*** | **B*** | **A*** | **-** | **B*** | A* | **A*** | **7** |
| Awadalla,H etal.201 | **A*** | **A*** | **-** | **A*** | **-** | **B*** | A* | **A*** | **6** |
| Bello,A. et al,2019 | A* | **A*** | **-** | **A*** | **A*** | **B*** | A* | **A*** | **7** |
| Ede, O.etal,2018 | B* | **B*** | **A*** | **A*** | **A*** | **-** | A* | **A*** | **7** |
| Jarso,G. etal,2011 | **A*** | **A*** | **A*** | **B*** | **A*** | **A*** | A* | **A*** | **8** |
| Jember,G.et al,2017 | **A*** | **A*** | **A*** | **A*** | **A*** | **B*** | A* | **A*** | **8** |
| Khalil,S.A.etal,2019 | A* | **B*** | **B*** | **A*** | **-** | **B*** | A* | **A*** | **7** |
| Kisozi,T. etal 2017 | B* | **B*** | **A*** | **A*** | **A*** | **-** | A* | **A*** | **7** |
| Kuate-Tegueu C et al,2016 | **B*** | **A*** | **A*** | **B*** | **A*** | **A*** | A* | **A*** | **7** |
| Mba IE etal 2001 | **A*** | **A*** | **A*** | **A*** | **A*** | **B*** | A* | **A*** | **8** |
| Mohamed, M.M etal 2019 | **A*** | **A*** | **A*** | **A*** | **A*** | **B*** | A* | **A*** | **7** |
| Mohmad AH et al 2011 | B* | **B*** | **A*** | **A*** | **A*** | **-** | A* | **A*** | **7** |
| Ogbera AO et al.2015 | A* | **A*** | **B*** | **A*** | **A*** | **B*** | A* | **A*** | **8** |
| Oguejiofor, O.C .et al,2019 | **A*** | **A*** | **A*** | **A*** | **A*** | **B*** | A* | **A*** | **7** |
| Ojieabu, W.A.etal,2016 | **A*** | **A*** | **A*** | **B*** | **A*** | **A*** | A* | **A*** | **8** |
| Olamoyegun M,etal,2015 | **B*** | **A*** | **A*** | **B*** | **-** | **A*** | A* | **A*** | **6** |
| Owolabi, M.O. Etal,2012 | A* | **B*** | **B*** | **A*** | **-** | **B*** | A* | **A*** | **7** |
| Tamba, S.M., et al,2013 | **A*** | **A*** | **A*** |  | **B*** | **B*** | A* | **A*** | **6** |
| Ugoya, S.O., et al, 2006 | **A*** | **A*** | **A*** | **A*** | **-** | **B*** | A* | **A*** | **7** |
| Worku,D.et al,2101 | **A*** | **A*** | **A*** | **A*** | **A*** | **B*** | A* | **A*** | **7** |

*Note: from each item account point. (Accept the study if total score ≥7)*

Selection: (Maximum 5 stars)
1) Representativeness of the sample: a) Truly representative of the average in the target population. * (all subjects or random sampling) .b) Somewhat representative of the average in the target population. * (nonrandom sampling) .c) Selected group of users.d) No description of the sampling strategy.
2) Sample size:a) Justified and satisfactory. *.b) Not justified.
3) Non-respondents: a) Comparability between respondents and non-respondents characteristics is
established, and the response rate is satisfactory. * .b) The response rate is unsatisfactory, or the comparability between respondents
and non-respondents is unsatisfactory. c) No description of the response rate or the characteristics of the responders and
the non-responders.
4) Ascertainment of the exposure (risk factor): a) validated measurement tool. ** .b) Non-validated measurement tool, but the tool is available or described.* c) No description of the measurement tool.
Comparability: (Maximum 2 stars)
1) The subjects in different outcome groups are comparable, based on the study design or analysis. Confounding factors are controlled. a) The study controls for the most important factor (select one). * b) The study control for any additional factor. *
Outcome: (Maximum 3 stars)
1) Assessment of the outcome: a) Independent blind assessment. **,b) Record linkage. **,c) Self report. *,d) No description.
2) Statistical test:a) The statistical test used to analyze the data is clearly described and appropriate, and the measurement of the association is presented, including confidence intervals and the probability level (p value). *,b) The statistical test is not appropriate, not described or incomplete
